# Method for the Analysis of the Tumor Microenvironment by Mass Cytometry: Application to Chronic Lymphocytic Leukemia

**DOI:** 10.3389/fimmu.2020.578176

**Published:** 2020-10-20

**Authors:** Susanne Gonder, Iria Fernandez Botana, Marina Wierz, Giulia Pagano, Ernesto Gargiulo, Antonio Cosma, Etienne Moussay, Jerome Paggetti, Anne Largeot

**Affiliations:** ^1^ Tumor Stroma Interactions, Department of Oncology, Luxembourg Institute of Health, Luxembourg, Luxembourg; ^2^ Faculty of Science, Technology and Medicine, University of Luxembourg, Esch-sur-Alzette, Luxembourg; ^3^ National Cytometry Platform, Quantitative Biology Unit, Transversal Activities, Luxembourg Institute of Health, Luxembourg, Luxembourg

**Keywords:** mass cytometry, chronic lymphocytic leukemia, lymphoma, microenvironment, immunosuppression

## Abstract

In the past 20 years, the interest for the tumor microenvironment (TME) has exponentially increased. Indeed, it is now commonly admitted that the TME plays a crucial role in cancer development, maintenance, immune escape and resistance to therapy. This stands true for hematological malignancies as well. A considerable amount of newly developed therapies are directed against the cancer-supporting TME instead of targeting tumor cells themselves. However, the TME is often not clearly defined. In addition, the unique phenotype of each tumor and the variability among patients limit the success of such therapies. Recently, our group took advantage of the mass cytometry technology to unveil the specific TME in the context of chronic lymphocytic leukemia (CLL) in mice. We found the enrichment of LAG3 and PD1, two immune checkpoints. We tested an antibody-based immunotherapy, targeting these two molecules. This combination of antibodies was successful in the treatment of murine CLL. In this methods article, we provide a detailed protocol for the staining of CLL TME cells aiming at their characterization using mass cytometry. We include panel design and validation, sample preparation and acquisition, machine set-up, quality control, and analysis. Additionally, we discuss different advantages and pitfalls of this technique.

## Introduction

In chronic lymphocytic leukemia (CLL), the microenvironment is crucial. CLL cells need to interact with their neighboring cells for their survival and proliferation. This is true for all the organs were CLL cells can be detected: in the blood, bone marrow, spleen, and lymph nodes. In humans, the very specific nodal microenvironment is an important site for activation of the B-cell receptor (BCR) and nuclear factor kappa-light-chain-enhancer of activated B cells (NF-KB), which directly drives cell proliferation and disease progression (1). The leukemic microenvironment (LME) is highly immunosuppressive, and these immune cells represent crucial targets for immunotherapy. However, the development of such therapies is limited due to the lack of knowledge in the composition of the LME and to the singularity of this environment for each cancer type. In addition, it is composed of a high variety of stromal and immune cells, which often display phenotypes exclusive to the LME, with the appearance of populations unique to this very specific environment. In this context, the use of high dimensional techniques is crucial to unravel the LME in its full diversity. Mass cytometry was developed in the late 2000s/early 2010s in order to push forward the number of cellular parameters that could be analyzed in parallel compared to conventional Flow Cytometry (FC) analysis. Whereas in the early 70s, the detection of a single color by FC was a revolution, it appeared clearly that conventional cytometry was unable to reach the contemporary requirement and the willingness to decipher highly heterogeneous cell populations. Whereas mass cytometry technology allows the analysis of up to 50 parameters in parallel, it is still associated with a relatively high throughput compared to conventional FC. This was made possible by combining FC platform with mass spectrometry analysis. Mass spectrometry is able to precisely separate unique stable metal isotopes based on their atomic weight. These isotopes can be coupled to antibodies and used to stain cells. Contrary to conventional fluorophores-based cytometry, virtually no overlap between the different isotopes is observed, which enables to have up to 50 parameters detected simultaneously presently. The number of parameters analyzed, combined with the high throughput give rise to many advantages, among those, the detection of rare cell subsets and the discovery of novel cell populations or sub-populations, as markers which would not have been analyzed together in a normal FC panel can now be grouped. It must be noted that novel flow cytometers (e.g., spectral flow cytometers) can detect a similar amount of parameters.

Recently, we took advantage of the power of mass cytometry to decipher the splenic LME of the Eμ-TCL1 mouse, the canonical model for CLL, which allowed us to identify specific immune cell populations and the immune checkpoints PD1 and LAG3 as potential targets in CLL (2). Other groups applied this technology to study either LME or directly the diversity of the leukemic cells themselves in the context of B cell malignancies (3–6). However, these studies are still very rare in this field. It is worth noting that mass cytometry is classified as a tier 1 assay for cell profiling in the Cancer Immune Monitoring and Analysis Centers (CIMACs), which highlights the advantage of this technique.

Here, we provide a stepwise protocol for the staining of cells prior to acquisition on a mass cytometer, preceded by explanations, tips and troubleshooting for panel design, samples preparation, acquisition, and analysis, which are useful for new users of this technology.

## Methods

The experimental design in mass cytometry requires thoughtful planning as some experimental factors can have an impact on the acquisition, data analysis and the results. These factors include panel design, sample preparation, storage, fixation, and sample stimulation. Thus, the verification and standardization of the assay is highly important to avoid misleading interpretations.

### Panel Design

The association of the antibody or probe with corresponding heavy-metal isotope to achieve the best possible signal intensity for the detection of specific targets on single cells is one of the most precious steps in the experimental design using mass cytometry (7). In general, the rules of panel design for FC are applicable to mass cytometry, with some adaptations regarding isotopic mass and contamination. Moreover, it is possible to adapt the antibody clones used for FC also for the mass cytometry panel as specificity and affinity to the matching antigens are already known.

**Figure 1:**
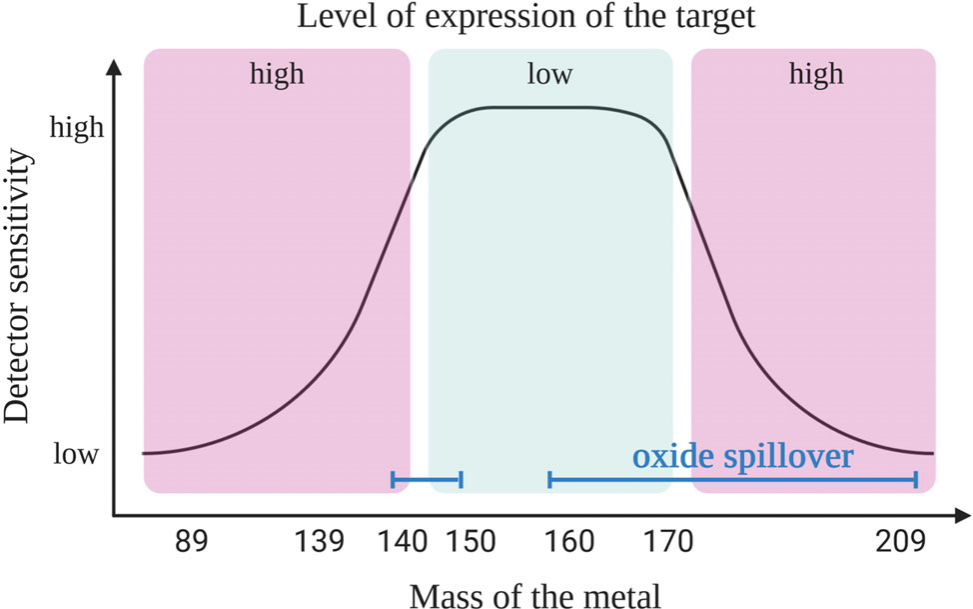
Coupling target with an appropriate heavy metal. Primary targets (high expression) should be labeled with heavy metal isotope-coupled antibodies that are detected with a lower sensitivity (low or high masses). The most sensitive detectors should be reserved for the tertiary targets (low expression level). Due to oxide formation the pairing of antibodies with isotopes is not as simple as shown here. Oxide interference (blue line) are seen in masses >157 Gd but also in the range of lanthanide elements (140s), and this should be taken into consideration when designing the panel, as explained in the main text. Created with BioRender.com.

First, it is crucial to understand the capabilities of the instrument. For example, the “Helios” version of the mass cytometry instruments is able to detect ions with atomic masses between 75 and 209 Da. It provides 135 channels out of which only approximately 50 are available today due to the current limitations of the heavy metal isotopes availability. In mass cytometry the sensitivity of the channels is dependent on the mass window of the detector which has a maximum sensitivity in the upper-middle channels (~157Gd to 170Er) (**Figure 1**). A combination of two factors explains the difference in sensitivity of the channels. First, because of collisions, ions with lower mass have more probability to be ejected from the ion beam during acquisition. Secondly, as ions with a mass-to-charge ratio (*m/z*) lower than 80 are filtered out (ions that are naturally found in the cells that need to be removed) and the quadrupole mass filter dedicated to this function has a maximum of efficacy for ions with a medium mass (8, 9).

In order to design a mass cytometry panel, the markers that need to be analyzed are first classified as primary (to identify major backbone subsets, such as CD45, CD3, CD4, and CD19; highly expressed and well defined), secondary (to fine-tune subsets, such as CD44, CD62L, CD23, and CD27; medium and variable expression) or tertiary antigens (low, unknown or variable expression, such as IL-10, LAG3, T-bet, and HELIOS). Then, the primary targets are associated to weak channels and the tertiary markers to the channels with the highest sensitivity. When designing the panel, it is important to have information on the expression level of considered marker in the specific context of the study. Indeed, the cell types, the type of sample and organs from where the cells originate may affect the level of expression for some markers. For example, the CD5 molecule is expressed on T and B cells including CLL cells; however, B cells usually express this molecule 2- to 5-fold less compared to T cells (10). In addition, CLL B cells show a high heterogeneity of CD5 expression (11) and patients with lower level of CD5 show a more aggressive disease (12). All these information will have an impact on choosing the correct channel for detection depending on the cell of interest. This first step of panel design needs adaptation according to the next steps described below, where three sources of crosstalk among channels need to be considered.

**Figure 2:**
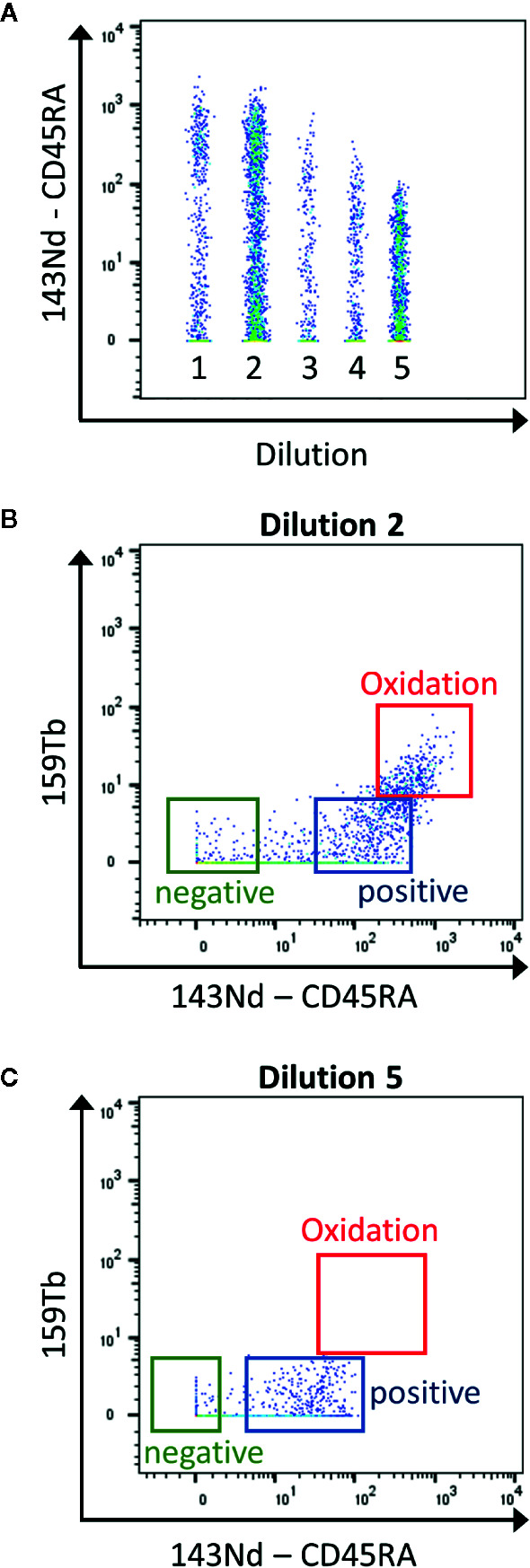
Determination of the contamination due to metal oxidation during the titration. **(A)** The samples are concatenated in order to display the 5 dilutions for the CD45RA antibody in the same graph (1 to 5: range from 1:1 to 1:81 dilutions) **(B)** Using the second dilution (1:3), the CD45RA antibody gives a signal in the empty 159Tb channel, corresponding to oxidation of the metal (red box). **(C)** This signal is clearly reduced using the 5^th^ dilution (1:81). This dilution will be used as it has the lowest contamination, still giving a good positive signal over negative one (Blue and green boxes).

Mass cytometry is used for the detection of ~30–50 markers as it has the advantage compared to classical flow cytometry, to have no spillover between the heavy metal isotopes used as labeling. However, it is important to know the sources of crosstalk between channels. First, “isotopic impurity” is a contamination of the heavy metal with one of its isotopes. Even though a 100% pure isotope preparation is desirable for the use in mass cytometry, this is not feasible for all the metals used. Secondly, “abundance sensitivity” is explained by an error in the detection of the ions during acquisition. Abundance sensitivity can be detected in the channels with a mass higher or lower of 1 (“M+1”, “M-1”). Finally, after ionization some metals have a tendency to oxidize, which might lead to an increase in the element mass by 16 (^16^O, “M+16”, **Figure 2**). La, Ce, Pr, Nd, and Gd metals have high levels of oxide formation (13). These oxidations cannot be eliminated but can be decreased by optimal setting of the make-up gas (14).

**Figure 3:**
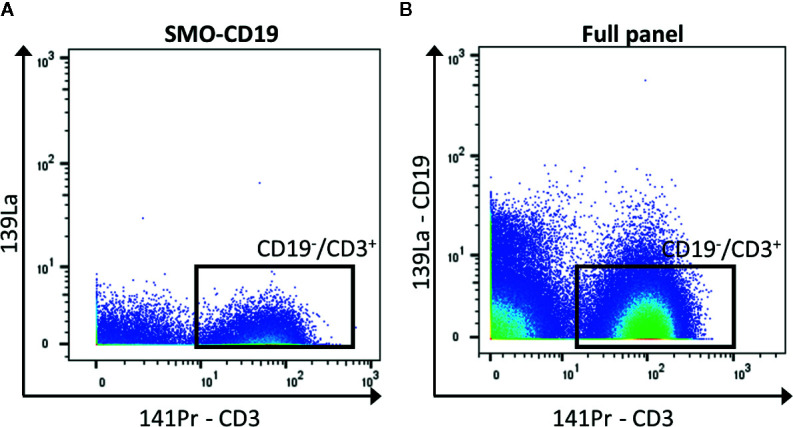
Exclusion of CD19^+^ cells. **(A)** The negative control for CD19 (SMO-CD19) is used to set the gate of the CD19^−^/CD3^+^ cells (black box). **(B)** The few remaining CD19+ cells are removed from the analysis by gating on CD19^−^/CD3^+^ cells in the full panel (black box).

In general, when possible, it is recommended to design a panel with heavy metals separated by a mass difference of more than 1, and to avoid metals with M+16 mass. For panels with a high number of detected markers, this is not possible. To reduce the crosstalk, markers that are less expressed can be selected for less-pure metals. Hence, the contamination of this isotope will be minimum. If these metal-labeled antibodies are selected for primary markers, avoid adding a tertiary target at the M+16 and M +1/−1 positions. In addition, it is recommended to select markers that label well identified cell subsets for less-pure isotopes as their M+16 and M +1/−1 contamination can be easily spotted (for example CD4 and CD8 are virtually not co-expressed outside of the thymus). Furthermore, in case of expected high contamination in one channel, the latter should be used for an exclusion marker (cells that need to be gated out, for example CD19+ cells if the interest resides in T cells, **Figure 3**).

Some strategies have been developed to correct any crosstalk between channels. Chevrier et al. have proposed a compensation method using a bead-based strategy and R-based software (15). This new method provides the first steps into mass cytometry compensation to avoid artifacts and improve sensitivity, although, a good panel design is still required to minimize crosstalk between channels.

**Figure 4:**
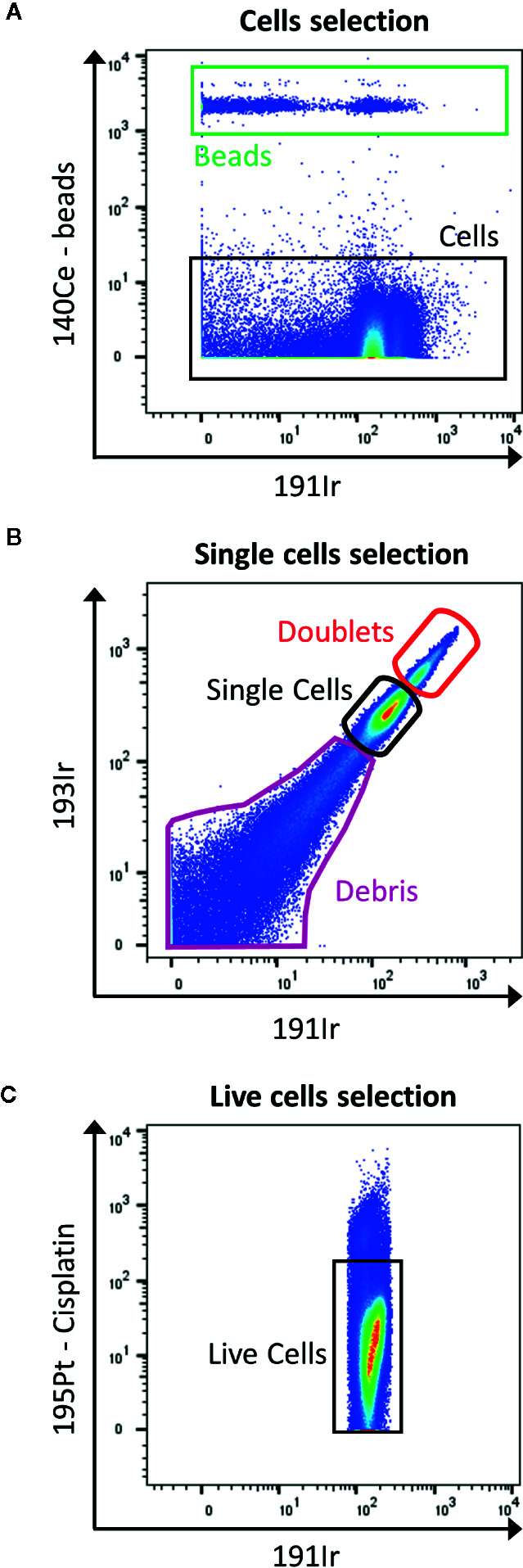
Gating strategy for live single cells events. **(A)** The calibration beads (red box) are gated out by selecting 140Ce^-^ population (black box). **(B)** Then, singlets are discriminated from doublets by displaying 193Ir vs. 191Ir (DNA intercalator). The single cells are the well-defined population in the middle part of the dot plot (black box). The population with a higher signal in Iridium are the doublets (red box), and the one with lower signal are the debris (purple box). **(C)** Finally, the live cells are gated as negative for cisplatin staining (black box).

In contrast to flow cytometry, cells are not detected by their size and granularity using forward scatter and side scatter as the cells are vaporized. In order to track cells, after permeabilization they are labeled with rhodium- (103Rh) or iridium-based (191Ir) DNA intercalators for nucleated cells (**Figure 4A**) and specific probes to characterize non-nucleated cells (16). In addition, the mass cytometer is unable to measure information of height, area and width as in the fluorescence FC to discriminate doublets from singlets. However, there are possibilities to reduce the amount of doublets by slowing down the acquisition rate, filtering and diluting the sample, bar coding and gating out events that show a higher DNA intercalator signal (**Figure 4B**). However, this limits the information available in term of DNA content of the cells, if the interest resides in cell cycle. In this case, 5-iodo-2-deoxyuridine (IdU; atomic mass of 127) detects cells which underwent DNA synthesis (17).

The viability staining is performed before the permeabilization step, by using cisplatin which enters the disrupted cell membrane of dead cells more easily than of live cells. Cisplatin binds nonspecifically to intracellular DNA and proteins and is detected in 195Pt channel (**Figure 4C**).

Having these rules in mind, the use of dedicated software is highly recommended to help for the design of specific panels. First, Fluidigm supplies already preconfigured screening panels for comprehensive phenotyping and functionality of certain cell populations, which will save time and simplify the experiment. Secondly, Fluidigm provides the online Maxpar Panel Designer (https://dvssciences.com) that calculates and visualizes predicted signal overlaps by the selection of available pre-conjugated antibodies from Fluidigm and custom antibodies/probes.

Heavy metal-labeled antibodies and kits are commercially available. However, they can be very limiting in regard to the panel design. This limitation demands in-house conjugating of the antibodies with the heavy metal-labeled isotopes. Guojun Han et al. provide a detailed protocol about conjugating antibodies with heavy-metal isotopes which requires different methods due to their chemistry and stability (18). Another method to overcome the issue of unavailability is the two-step staining. This consists in detecting the targets with primary monoclonal antibodies labeled with fluorophores followed by detection of this fluorophore by secondary antibodies conjugated with the desired metal isotopes.

In case of a very weak signal, it is possible to amplify it by either two-step staining or a second antibody that recognizes a different epitope on the target (14).

### Panel Titration and Test

After designing the panel, it is crucial to titrate the antibodies, test the full panel and the experimental workflow before moving forward to the final samples.

#### Titration of Antibodies

As in flow cytometry, the titration of heavy metal-labeled antibodies is the key to optimize staining conditions, reduce nonspecific binding of antibodies and to validate the contamination in M+/−1 and M+16 channels.

In general, a serial dilution strategy of at least 5 dilutions (up to 7) is recommended for the selection of the appropriate concentration. Based on our experience, we recommend testing also higher concentrations than those suggested from the antibody data sheets. In our laboratory, we mostly perform the titration with a 1:3 dilution factor. First, we titrate the antibodies of primary markers. Afterward, we perform the titration of the secondary and tertiary targets, expressed only on specific subsets, by adding the known concentration of the primary markers to gate the lineage populations (context titration). For example, after the titration of CD3, CD4, CD8, and CD19, the antibodies for FoxP3, CD25, PD1, and LAG3 can be titrated. This has two advantages: to titrate the secondary in the context of the population of interest, but also, to enrich the signal by gating the cells which are known to express the secondary/tertiary markers. For further information, Van Vreden et al. provide a detailed protocol for the titration of new antibodies (19).

If using probes after long-term storage (more than 6 months), it is important to re-titrate them. In addition, we experienced that storing the antibodies in an auto-defrost fridge induces a higher evaporation compared to a classic fridge, greatly affecting the concentration of the stock. Thus, we recommend storing the antibodies in a fridge without auto-defrost mechanism.

**Figure 5:**
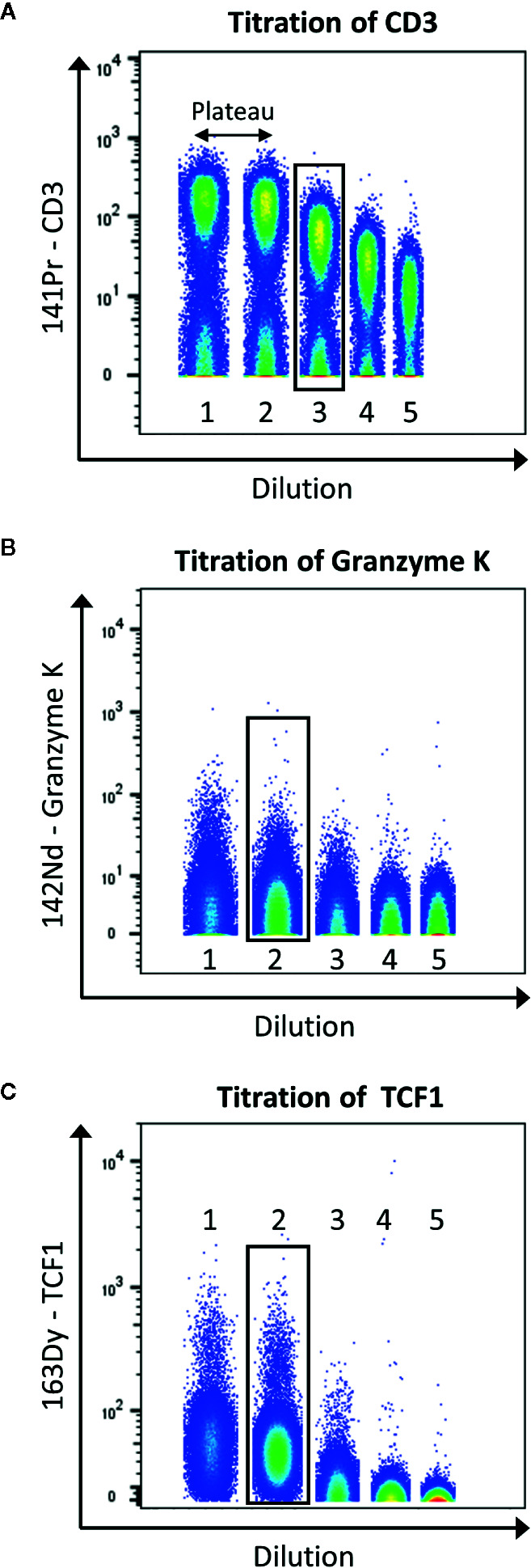
Example of titration for the antibodies anti-CD3 **(A)**, -granzyme K **(B)**, and -TCF1 **(C)**. Data were uploaded in Flowjo and for each titration sample, and singlets live cells were gated as described before. Then, samples were concatenated to display on the same plot the five dilutions (range from 1:1 to 1:81). The dilution showing the best separation between negative and positive signals, before the appearance of a plateau was selected (black box).

**Figure 5** displays the selection of the correct concentration for antibodies. Here, we use the tool to concatenate samples in Flowjo10 (Becton, Dickinson & Company) to display all the dilutions in one plot. There should be a good separation between the negative and positive population. To help define this separation factor, the staining index can be calculated. It is the ratio of the separation between positive and negative population divided by two times the standard deviation of the negative population (the higher, the better). If a plateau is observed, the first dilution before this plateau should be selected. In addition, it is also important to take into consideration the signal in the contaminated channels (M+16 and M+/−1). The selected dilution should have the better separation, before the appearance of the plateau, and giving as less contamination as possible.

#### Full Panel Test

After titration, testing the full panel on an appropriate control sample is essential. As some experiments use rare samples which are too precious to be used for the tests (BM or LN biopsies), the type of sample requires a careful consideration. For example, PBMCs from healthy donors are easy to obtain, however, stimulation or treatments should be considered, in order to induce expression of markers that are not found in PBMCs from healthy donors (such as exhaustion markers, specific cytokines, transcription factors…). In addition, these samples need to be handled in the same way as the experimental samples (e.g., isolation method, cell enrichment, storage…).

Assessing the impurity and oxidation rate of antibodies is also an important step during the full panel test. If it is not possible to reduce the contaminations by tuning the instrument or reducing concentration of the antibody, adjustments of the panel need to be considered.

During this test, we recommend to acquire an unstained sample, in order to validate the potential contamination due to reagents used for cell preparation. For example, barium is one of the most abundant elements, and is found in laboratory dish soaps. Distilled water can contain low levels of mercury, lead or tin or even iodine (14). Small adaptions in the daily laboratory life help to minimize the environmental contamination, such as using reagents with a high purity, new plastic or glass container for the storage of the buffers and testing these for contaminations on the mass cytometer. High purity reagents are commercially available.

### Controls Used in Mass Cytometry

#### Panel Controls

In flow cytometry experiments, isotype controls can be useful. Unfortunately, until now, isotypes with the corresponding metal are not commercially available (16). Fluorescence minus one (FMO) controls are used in high multiparameter flow cytometry to account for the residual spillover following proper compensation. In mass cytometry, the cross-talk between channels is minimal (see *Panel Design*) and can be further attenuated by proper panel design. Therefore the signal-minus-one or metal-minus-one controls (SMO/MMO, respectively) are useful for unknown markers (mainly tertiary ones) or for panels with sub-optimal design. In addition, metal-minus-many (MMM) can also be used, where several targets are subtracted to the main panel (16). An additional control that can be used to determine gate boundaries is to consider positive and negative cell populations within the sample. For example, outside of the thymus, virtually all CD4^+^ cells are CD8^-^ (14). When using a two-step staining, it is necessary to include controls such as secondary antibody with and without a primary antibody, in order to examine the unspecific binding properties of the secondary antibody (14).

#### Control to Reduce Variability During Longitudinal Studies

**Figure 6:**
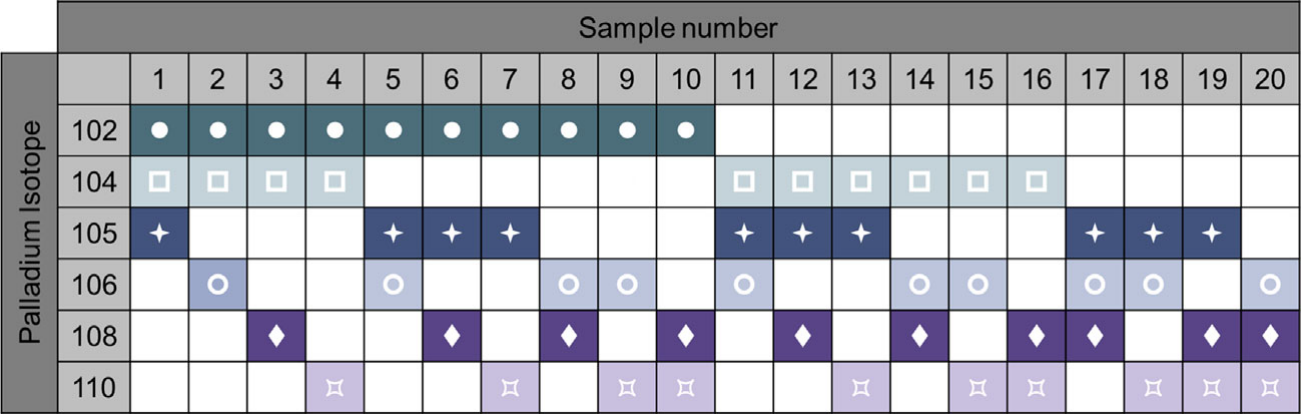
Example of 6-choose-3 barcoding matrix with 6 palladium isotypes. Heavy metal-labeled cellular barcoding enables running multiple samples in one tube by staining each sample with a unique combination of isotopes before pooling the samples. Here, up to 20 samples can be combined by different staining pattern. Following oftware debarcoding, samples can be analyzed individually. For example, sample 1 will be positive for Palladium 102, 104, 105, but negative for the 106, 108, and 110 isotopes. Adapted from Fluidigm.

Sample barcoding of the samples is a strategy that increases the efficiency of the process and decreases technical variability. Heavy metal-labeled cellular barcoding enables running multiple samples (multiplexing) in one tube by staining each sample with a unique combination of isotopes before pooling the samples. Bodenmiller and colleagues published a method for barcoding (20). A probe, which recognizes thiol groups in the cells (maleimido-mono-amide-DOTA (mDOTA), non-specific labeling of cells), is conjugated with 7 different lanthanide isotopes. This results into 128 (2^7^) possible combinations. Zunder et al. further improved this method (21). Indeed, lanthanide can be conjugated to antibodies, thus the previous barcoding method restricts the use of such antibodies. Then, lanthanide are a source of contamination (abundance sensitivity) and oxidation. Here, they used palladium isotopes, which cannot be used for antibody conjugation (chemical incompatibility with the conjugation tools) so far, and have limited oxidation rate. In this method, they chelated 6 palladium (Pd) isotopes with isothiocyano-benzyl-EDTA, which labels proteins through their amine groups, allowing 64 combinations. Accordingly, the Cell-ID 20-Plex Pd is commercially available by Fluidigm providing 20 combinations (**Figure 6**). A drawback of these protocols is the need of staining on fixed and permeabilized cells. Lai et al. developed a multiplexing protocol using CD45 to barcode PBMCs (22). As the result of the acquisition of multiplexed samples, it reduces technical and instrumental variations. This being said, it is important to keep in mind that if one or more low-quality samples (poor viability, high amount of debris) are added in the pool of samples, the background will increase, reagent titer problems will occur, and the recovery can be reduced due to sample clumping, jeopardizing all the samples (7). Pipetting errors of the large antibody panels is an important source of variability. Therefore, lyophilization of the antibody mixes can be adopted (23).

For longitudinal studies, which involve separate acquisition of samples that need to be compared and cannot be pooled, it is important to implement a technical control. In this case, three types of controls are suggested and combination of those is possible. First, the use of a unique sample that will be stained and acquired at every run of acquisition is advised. This requires freezing of enough aliquots to cover the period of the study and will allow taking into consideration the variability in the staining. Another method is to include labeled spike-in samples as an internal reference to monitor variability between batches concerning the machine itself. These spike-in controls can be labeled with an isotope which is usually not used in the analysis of mass cytometry, such as Tantalum (23). Finally, Polystyrene bead standards containing known concentrations of the metal isotopes are used to normalize data using a mathematical algorithm that corrects for decrease in sensitivity of the instrument over the time of the study (further discussed in *Acquisition*).

### Sample Preparation and Staining

Even though clinicians perform the sample collection of patient material and thus it is often not controllable, it is crucial to develop a clean sample preparation for a reliable data acquisition and analysis. Testing different protocols on healthy donor material before moving forward to the actual precious samples is highly suggested if not already established in the laboratory. Leelatian and colleagues published different protocols generating viable single cell suspension derived from human peripheral blood (24) and from a variety of human tissues and tumors (25) which are useful to obtain samples with a high cell viability and little amount of debris.

It is often necessary to enrich the samples for the cell of interest. For example, in the case of CLL, if the focus of the study lies on T cells, CD19^+^ depletion to remove CLL cells is advised, whereas, if the leukemic cells are the one to study, negative selection of CD19^+^ cells is preferred. The idea is to leave the cells of interest untouched. If considering a pre-enrichment with magnetic beads it is important to choose one that does not interfere with the detection of the mass cytometry. Thus, testing the reagents for background detection on the mass cytometer is recommended. In our experience, the use of the MACS Cell separation system (Miltenyi) does not interfere with the detection on the mass cytometer. Other methods to enrich cell populations are Fab-based Traceless affinity cell selection (IBA lifesciences) and fluorescence-activated cell sorting. In any case, we suggest to include antibodies in the panel that target cells which should have been removed by the pre-enrichment step, to be able to exclude the remaining cells during the analysis (**Figure 3**). If the sort consist of negative selection using multiple markers (for exclusion of T cells: CD3, CD4, and CD8), it is possible to use several antibodies with the same labeling isotope. Nevertheless, the user must be aware that cell enrichment will necessarily distort the frequencies of the different populations within the sample and can have an impact on the results. Consequently, adopting or not an enrichment step should be considered based on the biological question. An advantage of traditional FC over the CyTOF technology is its lower acquisition time. As a result, fluorophore-based cytometers can generally acquire larger samples without the need of enriching the population of interest.

**Figure 7:**
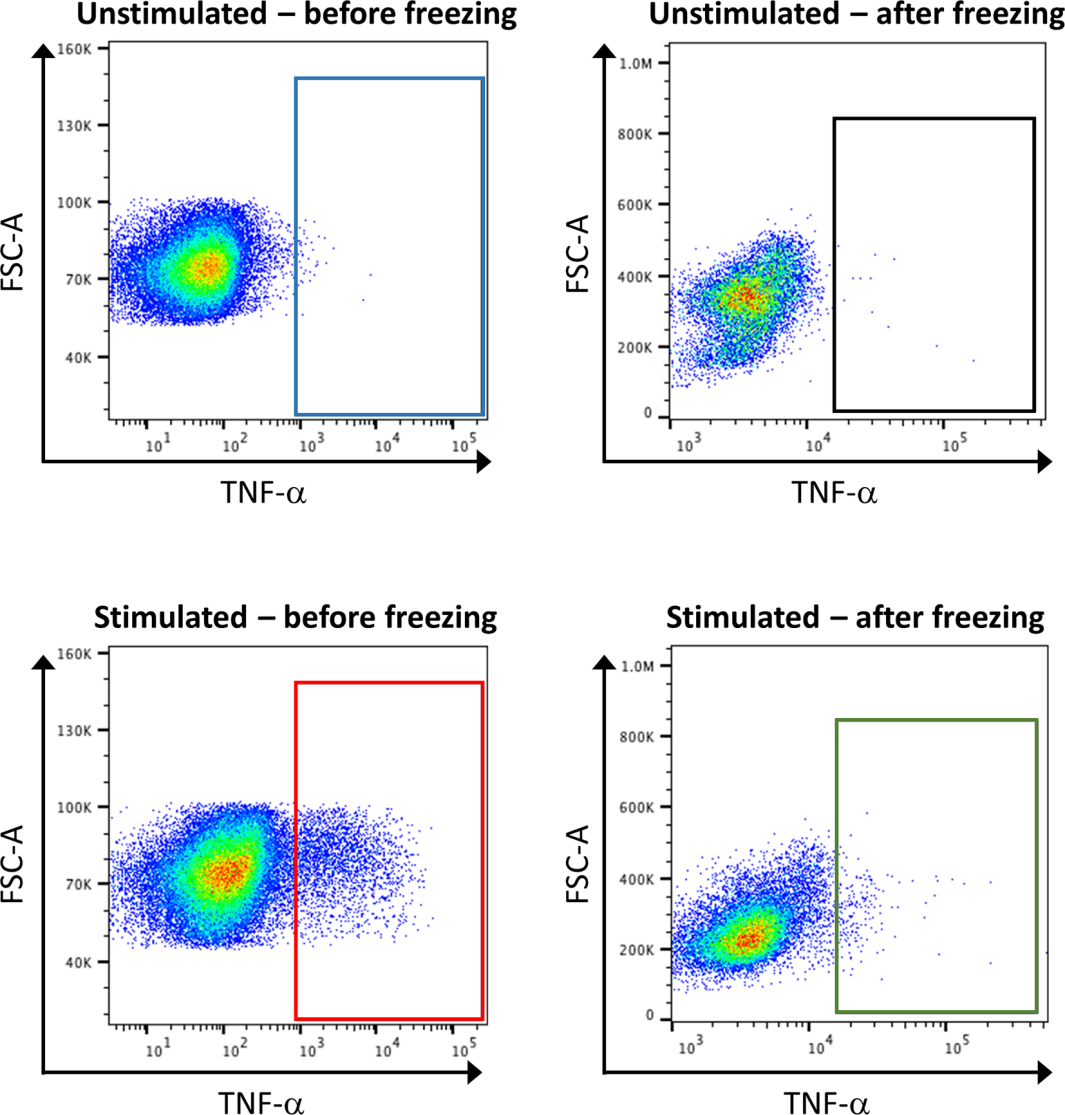
Consequence of stimulation and sample freezing on cytokine production and detection. Healthy control PBMCs were stimulated or not with PMA, and either directly subjected to classical FC, or frozen for several day before being subjected to FC. The analysis of TNF-α expression shows that stimulation leads to an increase of its expression (blue box vs. red box). However, the signal is lost after cell freezing (green box vs. red box).

Concerning sample handling, it is important to have in mind that freezing steps can greatly affect the expression of some markers, especially cytokines. Thus, it is recommended to test the effect of freezing on targets by conventional FC beforehand (**Figure 7**). If required, stimulation of the samples, by PMA and ionomycin, can be performed shortly before the staining. In order to hinder the transportation of the produced cytokines outside the cells, a reagent that stops the Golgi activity has to be added after stimulation, for example brefeldin A. However, the stimulation of the samples is artificial, and will reveal the maximum capacities of a cell to produce cytokines, but does not necessarily reflect this production in the microenvironment that would have been detected before freezing.

As for classical FC, it is recommended in mass cytometry experiments to use blocking reagents to reduce the staining of antibodies *via* their constant Fragment crystallizable region (Fc) domain on Fc receptors, which are mainly found on monocytes, macrophages, dendritic cells and B cells. However, considering other blocking methods is important if the CD16 or CD32 markers are of interest.

Extracellular staining for mass cytometry can be performed at room temperature, as it appears that internalization of antigens does not change the detection on the mass cytometer (which is important for flow cytometry, where staining is usually performed at 4°C). However, one should consider performing the fixation and permeabilization as recommended when using commercially available kits, which is mostly done at 4°C. After the surface and intracellular staining, the cells are incubated with the intercalator which will allow the cell detection. Before acquisition, it is possible to store the samples in the fridge for up to one week in the intercalator buffer. However, it is recommended to inject the samples to the mass cytometer as soon as possible, because long-term storage can have an effect on the detection of markers.

For storing of the samples, the use of polystyrene tubes/plates is preferred, however the recovery of cells on the mass cytometer is higher in polypropylene tubes. Thus, it is recommended to filter the cells into a polypropylene tube just before acquisition or for their storage.

Concerning the number of cells to prepare, it is important to know that in mass cytometry, only 50%–60% of the sample can be recovered, the rest of the sample will be lost due to the aggregation on the walls of the spray chamber and injector (7). An additional cell loss of 20%–30% should be taken into account as cells will be lysed and lost during the sample preparation, staining procedure and washing steps. Performing the staining and washing steps in a 96-well plate helps to reduce cell loss. In addition, resuspending samples in high purity water removes any contamination before acquiring the samples. Here, also a commercially available running buffer can be used to reduce cell breakdown and antibody dissociation (13).

### Acquisition

The CyTOF machinery is an inductively coupled plasma (ICP) time-of flight (TOF) mass spectrophotometer (MS). Samples are injected into the mass cytometer, manually or *via* an auto-sampler, and introduced into a nebulizer through a narrow capillary. Once in the nebulizer, the cell suspension is aerosolized into single-cell droplets by argon gas-based pneumatic nebulization and released into the spray chamber. Argon gas (also known as make-up gas) transports the cell droplets to the ICP torch along the heated spray chamber, subsequently shrinking them by evaporation. The sample introduction system has a cell transmission efficiency of approximately 60%–70% (7). Cells are then delivered into the plasma core wherein they are atomized, and the metal ions ionized, leading to the formation of a cloud of charged metal ions corresponding to single cells. The ion cloud passes through a quadrupole filter which removes low mass ions (m/z < 80) derived from naturally found elements in cells, such as carbon and oxygen, while allowing the flow of ions of analytical interest to proceed to the TOF chamber. Here, the reporter ions are accelerated at a fixed potential and thus travel at a speed proportional to the square root of their masses. Each mass ordered ion pulse is detected by an electrode multiplier, and the resulting signal recorded as a digitalized waveform by the detector *via* an analog-to-digital converter.

Continuous operation of the CyTOF machinery leads to loss of sensitivity due to several factors; including and build-up of cellular debris in nebulizer and cones. Even regular cleaning and maintenance can cause significant day-to-day signal variation. This is why it is crucial to use appropriate controls during longitudinal studies (see *Control to Reduce Variability During Longitudinal Studies*).

### Data Analysis

As discussed in previous sections, mass cytometry emerged as an alternative to traditional fluorescent-based flow cytometry, enabling the simultaneous detection of over 50 cellular parameters by using heavy metals as antibody/probes labeling reagents, and hence avoiding fluorescent spectral overlap.

The exponential increase in phenotypic and functional characteristics that can be analyzed, allows for the dissection of cellular diversity and heterogeneity with unprecedented resolution, and favors the discovery of new cell subpopulations by visualizing previously inaccessible marker combinations. The ability to perform such detailed single-cell profiling is particularly valuable for the dynamic characterization of immune cell subsets within the TME. This holds true for a wide range of solid and hematological tumors, including, as per our experience, B cell malignancies. However, the elevated number of parameters analyzed in parallel requires careful data processing in order to fully benefit from this technology. Here, we give a brief review of different analysis options, which, will provide the novel mass cytometry users with a better understanding of the distinct possibilities available. For further reading, more detailed reviews on data analysis are published by Kimball et al. and Pedersen and Olsen (26, 27).

#### Manual Gating

Mass cytometry data is saved as standard flow cytometry FCS 3.0 format, and consequently can be analyzed using traditional flow cytometry data analysis software such as FlowJo. In fact, manual gating can provide key insights on cellular abundance and expression and is particularly useful when it comes to user guided data analysis. However, interpretation of such complex data *via* manual gating of bivariate plots can become an overwhelming task, as the number of parameter pairs increases exponentially with the number of parameters analyzed. Additionally, meaningful multivariate relationships are lost as they cannot be discerned in two dimensions, and unanticipated cell populations can be unintentionally excluded given the subjective nature of manual gating. The immense complexity of the data generated demands exhaustive organization. Structured and consistent file nomenclature, as well as appropriate data storage, greatly facilitates downstream data processing and analysis. We recommend using manual gating for validation of the experiment (e.g., looking at specific cell populations that are known to be enriched or lost during treatment or between samples), to clean-up the data (gate out beads, dead cells, doublets of cells) and to exclude non-relevant cells (e.g., CD19^+^ cells if interest resides in T cells) before proceeding to algorithm-based analysis.

#### High Dimensional Data Analysis Using Algorithms

Different algorithms were developed during the past decade for high-dimensional data. Some of the most widely used software kits include viSNE (28), SPADE (29), Phenograph (30), Citrus (31), and X-shift (32). These computational tools use a variety of languages (e.g., Mathlab, R or Python), clustering methods (e.g., parametric vs. non-parametric), and dimensionality reduction approaches to generate a comprehensive depiction of multiparametric single cell measurements. Nevertheless, the data retrieved is highly impacted by the choice of the algorithm, which, as a result must be chosen based on the nature of the biological question.

Even though these algorithms are developed by bioinformaticians at the forefront of mass cytometry research, their use is directed toward bench scientists working on a wide variety of fields. viSNE and SPADE were among the first algorithms developed for multiparametric data analysis, and, in our experience, represent a user-friendly option for recent adopters of mass cytometry. After gating on the population of interest, the data selected are exported and uploaded to a cloud-based computational platform or to a specific software (such as Cytobank or Cytosplore, respectively). These allow the user to run several algorithms to interpret high-dimensional data, including viSNE, SPADE, Citrus, and FlowSOM. The user can run them in the Cytobank platform or as single standing algorithms using the respective R packages or Bioconductor-based tools (e.g., Cytofast) (33). We hereby present a brief introduction to viSNE and SPADE followed by a practical guide on CyTOF data analysis using these algorithms in the context of LME analysis in B cell malignancies.

viSNE is a nonlinear dimensionality reduction technique based on the t-distributed stochastic neighbor embedding (tSNE) algorithm which enables the analysis of high-dimensional data on a two-dimensional map. The resulting viSNE plot, reminiscent of a traditional scatter plot, shows a continuum of cellular phenotypes distributed by the parameters tSNE1 and tSNE2; wherein phenotypically related cells cluster together leading to the formation of phenotypic islands. viSNE maintains single-cell resolution and takes into account high-dimensional similarities between all cell pairs, providing information about nearby and distant cells while preserving the geometry and non-linearity of the data. Cells are colored according to the expression of a chosen parameter in order to identify the cellular identity of the island by co-expression of lineage markers. When analyzing a complex mixture of cells, we find particularly useful the creation of a grid of viSNE plots organized per sample for different lineage defining parameters. Manual gating within the viSNE plots can be performed while in cytobank to obtain information such as population cell number and percentage in each experimental sample.

The global overview of the sample provided by viSNE facilitates the identification of known cell types, the distinction of phenotypic diversity within these populations, as well as the discovery of unexpected cell subsets. One of the main limitations of viSNE is the need for random down-sampling of cells to avoid event overcrowding in the 2D scatter plot. With a limited number of cells to be displayed, cell number can be reduced equally or proportionally, depending on the similarities in event number among the different samples. Finally, it is important to consider that independent viSNE runs on the same dataset will produce different plots, and, as a result, it is only possible to compare experimental groups when subjected to the same viSNE run.

SPADE (Spanning-tree progression analysis of density-normalized events) is another unsupervised clustering algorithm which allows the visualization of high-dimensional single cell data as a 2D minimum spanning tree of interconnected nodes. Each node comprises a group of phenotypically related cells, with the size of the node indicating cell number, and the color quantifying the median intensity of a parameter of interest. The number of nodes in the SPADE tree is set by the user based on the expected cell populations to be found within the sample. We recommend launching the algorithm several times for each parental population using a different node number in order to find the most appropriate settings for the experiment in question. One aspect that must be taken into consideration is that the relative distance between two nodes is not proportional to the phenotypic similarities or differences among these populations, and thus, the SPADE tree structure can be slightly modified to suit the needs of the user. The specific phenotype of each cluster can be further characterized by creating a heat map including lineage marker MSI values derived from the multiparametric analysis. MeV, an open multiomics experimental viewer, is available for this purpose.

The schematic visualization of multiparametric data provided by SPADE facilitates the analysis of cellular heterogeneity and is particularly helpful when assessing changes in population structure. Nevertheless, given the agglomeration of events in nodes of phenotypically related cells, single cell resolution is lost.

In our experience, running both algorithms for the same dataset provides key complementary insights in order to analyze and interpret complex immunological data, viSNE gives a general overview of the high dimensional data while SPADE assesses any changes in population structure or marker expression within distinct cellular phenotypes.

The development of software kits for multiparametric data analysis is a rapidly evolving field. An increasing number of algorithms, which introduce novel visualization methods and overcome previous software restrictions are now being published and made available. In our laboratory, we continuously review and use new algorithms of interest as a complementary tool to viSNE and SPADE. A recent example would be H-SNE (Hierarchical Stochastic Neighbor Embedding), an algorithm available in Cytosplore software (34). H-SNE is able to represent multiparametric single cell data while maintaining non-linear relationships and, unlike viSNE, is not affected by overcrowding and hence not limited by cell number. A number of novel clustering algorithms have also been developed, and provide valuable insights when launched alongside SPADE. X-shift, for example, is a population finding algorithm available in the online platform VorteX. This algorithm utilizes multiparametric data to construct a weighted k-nearest-neighbor density estimation (kNN-DE) graph, followed by clustering based on cell event density (32). However, unlike SPADE, X-hit finds the optimal number of clusters in a data-driven manner. This reduces the potential over or under fragmentation of cell populations resulting from the estimated number of nodes when using SPADE. A common approach involves the integration of both algorithms: X-shift is used initially to define the number of cellular phenotypes, followed by the generation of a SPADE tree with an informed number of nodes.

#### Data Visualization

Downstream data analysis of CyTOF data can be performed using different software kits. Excel is commonly used for the analysis of single cell cytometry data, and hence is applicable to CyTOF data as well. However, the immense complexity of multiplex single cell technologies renders this method archaic and practically prohibitive. In order to effectively visualize the data, we find that Tableau, a data visualization tool, is the ideal program for downstream processing and visualization of high-dimensional CyTOF data (35).

For this purpose, Tableau offers two separate software kits, Tableau prep and Tableau desktop, designed for data preparation and visualization respectively. Initially, the data generated after running the high dimensional algorithms of interest are exported as a text file from the online platform or software (Cytobank, Cytospore). The text files are then imported to Tableau prep, which enables the user to combine, shape, and clean up data prior to analysis and visualization. Tableau Prep provides the data in a visual way by showing a row and column profile—whereas rows show every single cell and columns illustrate markers, sample ID- and further it displays every step during data preparation. The output of Tableau Prep is a table of the high-dimensional data organized in a format that fit to the researchers needs. Once the data has been appropriately prepared, it is exported to Tableau desktop, which allows visual analytics and data exploration and thus gives quick answers to specific research question (e.g., differences in expression of one marker between different conditions). The software is user-friendly and does not require advanced computational skills. With Tableau desktop, one can create and display different features, such as: create groups (e.g., healthy vs. tumor) and heatmaps, implement calculations with existing data (e.g., calculate the frequencies of cluster/total), display information (e.g., MSI values, cluster frequencies) and rebuild dot plots, viSNE plots and SPADE trees. Regarding the statistical analysis, Tableau desktop can be used to perform descriptive statistics (e.g., mean, median, percentile, standard deviation…). Nevertheless, complex statistical operations are not readily available. As a result, we easily export the needed data and perform our advanced analysis using statistic software such as Graph Pad Prism.

### Stepwise Procedure for the Staining

**Figure 8:**
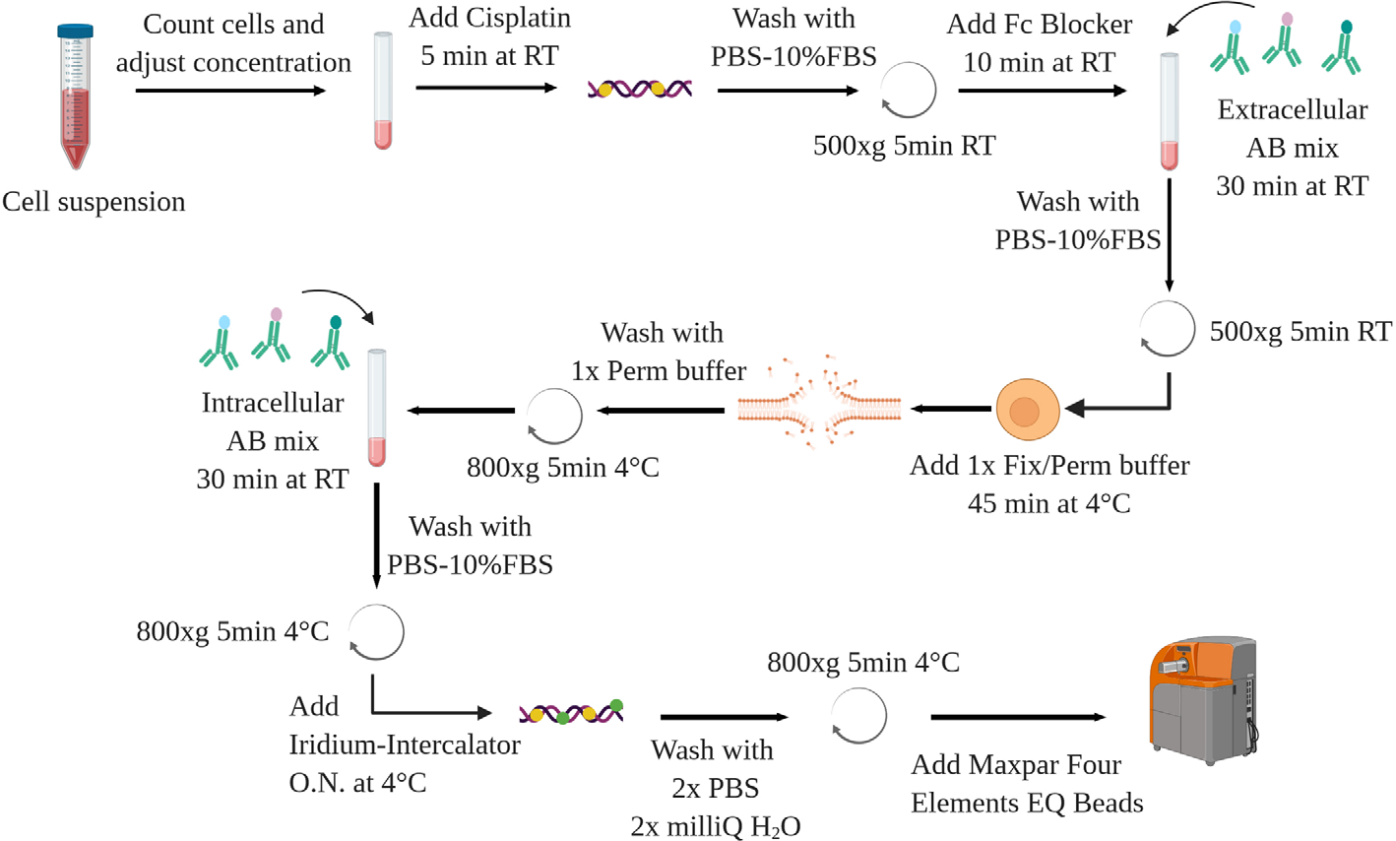
Schematic overview of the staining protocol. AB, antibody. Created with BioRender.com.

The following protocol is applied to murine splenocytes from control and leukemic (Eu-TCL1) mice. Isolation and purification of cells of interest are not a topic of this methods article. Chosen methods should ensure cell viability and preserve antigen integrity as for FC. The following can be performed on fresh or previously frozen cells, some restrictions applying to the latter (see *Sample Preparation and Staining*). A schematic overview is shown in **Figure 8**.

1. If required, thaw your cells in a water bath (37°C)
2. Transfer the cells in 10 ml of preheated Fetal Bovine Serum (FBS, Sigma Aldrich) or full media in a 50ml conical tube
3. Centrifuge at 500 x g for 5 min at room temperature (RT)
4. Remove supernatant (by discarding)
5. Resuspend cells in 1 ml of phosphate-buffered saline (PBS, without Mg^2+^/Ca^2+^ Life Technology) containing 10% of FBS (PBS-10% FBS)
6. Prepare a dilution and count the cells
7. Transfer the desired amount of cells (typically between 5.10^5^ and 3.10^6^) in a 15-ml conical tube, wash by adding PBS
8. Centrifuge at 500 x g, 5 min, RT
9. Re-suspend the cells in 100 ul of 5 µM Cell-ID Cisplatin (Cis-Pt, Fluidigm)
10. Mix and incubate for 5 min at RT
11. Wash with 5 volume of PBS-10% FBS, centrifuge (500 x g, 5min, RT) and discard supernatant
12. In the meanwhile, prepare extracellular antibodiy mix
13. Re-suspend the cells in 45ul PBS-10% FBS with 5ul of Fc Blocker (purified anti-mouse CD16/32 antibody, Biolegend)
14. Transfer 50µl of cell suspension in U-bottom 96-well plate (Corning)
15. Incubate for 10 min at RT
16. Add extracellular antibody mix (50 µl/sample)
17. Pipet up and down the samples and incubate for 30 min at RT
18. Stop the reaction by adding 100 µl of PBS-10% FBS, centrifuge 500 x g, 5 min, RT and discard the supernatant by reverting the plate
19. Wash by adding 200 µl PBS-10% FBS per well, centrifuge 500 x g, 5 min, RT and discard the supernatants
20. Add 100µl of 1× FoxP3 Fix/Perm buffer (from FoxP3/transcription factor staining buffer set, eBioscience) per well
21. Pipet up and down and incubate your samples for 45 min at 4°C
22. Meanwhile, prepare the intracellular antibody mix
23. Wash each well with 100 µl of 1× Permeabilization buffer (from FoxP3/transcription factor staining buffer set)
24. Centrifuge (800 x g, 5 min, 4°C) and discard supernatant
25. Re-suspend cells in 50 µl of PBS-10% FBS
26. Add the intracellular antibody mix (50 µl/sample)
27. Incubate the samples for 30 min at RT
28. Add 100µlper well, centrifuge 800 x g, 5 min, 4°C and discard supernatant
29. Wash the cells by adding 200µl PBS-10% FBS, centrifuge 800 x g, 5 min, 4°C and discard supernatant
30. Repeat step 28
31. In the meanwhile, prepare a solution of 50 nM Cell-ID Intercalator-Iridium (Fluidigm) in Maxpar Fix & Perm buffer (Fluidigm)
32. Re-suspend cells in 200 µl of the Iridium-Intercalator solution
33. Pipet up and down and incubate overnight at 4°C.
34. The next day, centrifuge the 96-well plate at 800 x g, 5 min, 4°C, and discard supernatant
35. Wash cells by adding 200 µl of serum/protein free PBS, centrifuge (800 x g, 5 min, 4°C), and discard supernatant
36. Repeat step 34
37. Wash the cells with 200 µl of milliQ water, centrifuge (800 x g, 5 min, 4°C), and discard supernatant
38. Repeat step 36
39. Re-suspend the cells in 200 µl of milliQ water and transfer through the cell strainer of the Round-Bottom Tubes with Cell Strainer Snap Cap (Falcon) into polypropylene tubes
40. Wash the cells with 200 µl of milliQ water and add on the filter
41. Repeat step 38 with 100 µl of milliQ water, turn the filter and take the residual volume (final volume in the 5ml round bottom tube should be 500 µl)
42. Determine the cell number
43. Adjust the volume to 90% of final volume (final concentration 1.5 × 10^6^ cells/ml: 900 μl for 1.5x10^6^ cells)
44. Add 10% Maxpar Four Elements EQ Beads (Fluidigm) before acquisition (for 1.5 × 10^6^ cells 100 μl of beads)

## Expected Results

It is widely admitted that the microenvironment plays a crucial role in the development and maintenance and progression of leukemia. However, in the context of CLL, this microenvironment is still understudied. In this context, we decided to decipher the immune cell landscape in a murine model of CLL, using mass cytometry few years ago (2, 36). We performed adoptive transfer (AT) of splenocytes from terminally diseased Eµ-TCL1 mice, which overexpress TCL1 exclusively in B cells and develop CLL within 8 to 12 months, into C57BL/6 recipient mice. Few weeks after the transfer, the recipient mice (AT-TCL1) developed the leukemia. The splenocytes from these diseased mice and from healthy control C57BL/6 mice were then subjected to CyTOF staining with a custom panel of 35 antibodies, following the above protocol. This panel was designed to study T lymphocytes, myeloid cells, and associated immune checkpoints. Following acquisition, the single live CD19^−^ cells were selected (manually gated) using FlowJo. Data were reanalyzed for the purpose of this methods article, therefore cluster numbers and number of associated cells differs from our original report (2). The respective FCS files were uploaded to Cytobank and SPADE was launched using all the markers except the one used for the initial gating strategy, with a target number of 50 nodes and 10% targets events downsampling. Then, viSNE was run on the SPADE data using the same markers, a maximum of 1.3 millions of cells, with the following parameters: iteration of 2,000, perplexity of 30 and Theta of 0.5. The data were then exported from Cytobank, uploaded to TableauPrep where they were organized, and subsequently analyzed with TableauDesktop.

**Figure 9:**
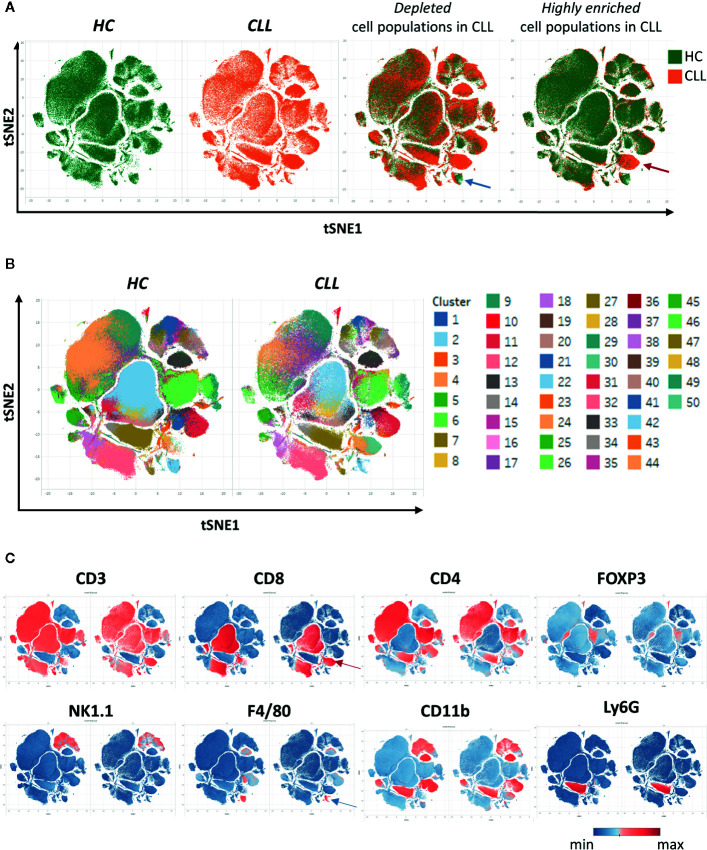
Clustering of cells using SPADE and viSNE, visualized through the Tableau software. **(A)** The viSNE plots are displayed for each sample separately (first and second panels). On the third and fourth panels, the overlay of the plots is shown, displaying the same amount of cells in each sample, which allows to easily detect the clusters which are depleted (e.g., blue arrow) or enriched (e.g., red arrow) in the CLL samples. **(B)** In order to identify the clusters, each one was associated to a color and displayed on the plot. **(C)** The intensity of the different markers are displayed, allowing to quickly get insights into the identity of each cluster. Here, the cluster enriched in CLL identified in **(A)** expresses CD8 (red arrow), and the one depleted (blue arrow) expresses F4/80.

**Figure 10:**
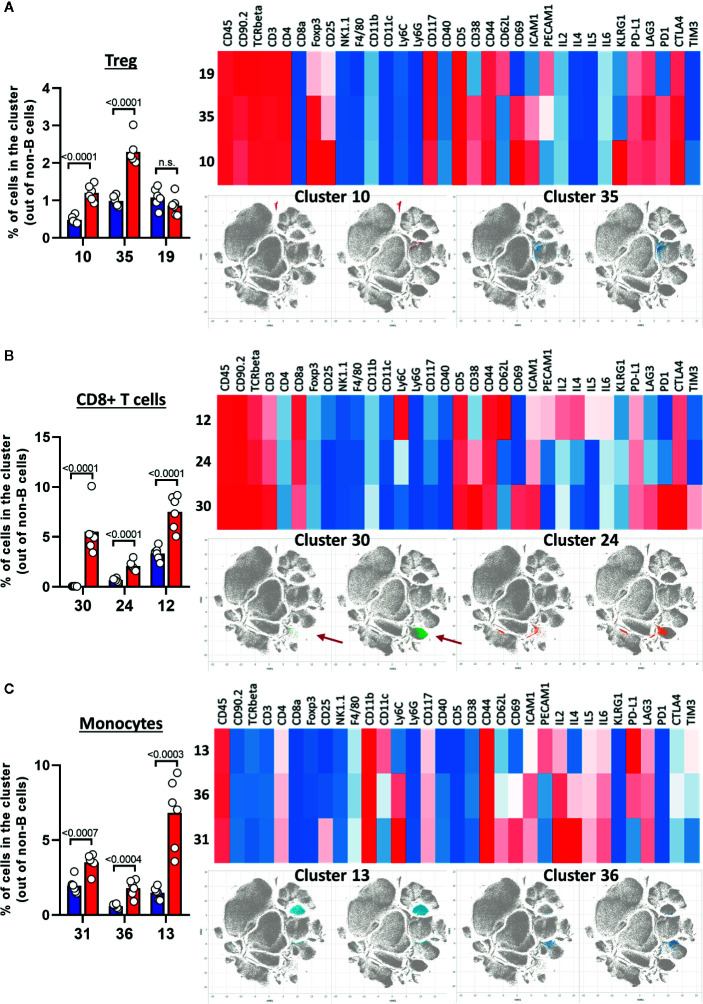
Characterization of cell populations enriched in CLL. **(A–C)** The left panels show the percentage of clusters in HC and CLL samples. The upper right panels show the heatmap for the expression of selected markers, the lower right panels highlight the position of enriched clusters on the viSNE plots. **(A)** Treg clusters. **(B)** CD8+ T cells clusters. **(C)** Monocytes clusters.

After creating two groups (healthy control HC and CLL samples), the viSNE plots can be visualized for the two groups separately. In addition, the plots for the two groups can be overlaid in order to easily identify cell populations which are highly enriched (red arrow) or depleted (blue arrow) in the CLL samples (**Figure 9A**). By allocating colors to each Spade cluster, they can be visualized on the viSNE plot (**Figure 9B**). In order to easily and quickly understand the composition of the viSNE plot, the expression of each marker can be displayed. This allows to get familiarize with the plot, and to know where the main cell populations sit (CD4+ T cells, CD8+ T cells, NK cells…). The cluster which was highly enriched in the CLL sample (**Figure 9A**, red arrow) is expressing high levels of CD8 (**Figure 9C**), whereas the one which was highly depleted (**Figure 9A**, blue arrow) expresses F4/80 (**Figure 9C**). After this first step in the understanding of the viSNE plot, the percentage of each cluster can be calculated in order to identify the clusters which are either enriched or depleted (**Figures 10A–C**, left panels). In addition, the MSI of each marker for each cluster can be extracted, and corresponding heatmaps can be created (e.g., in excel by conditional formatting) in order to define each cluster (**Figures 10A–C** upper right panels), which finally allows to precisely define each cluster, based on the expression of all the markers. Finally, the clusters can be highlighted on the viSNE plots (**Figures 10A–C**, lower right panels). In **Figure 10A**, three different clusters of Tregs are depicted, showing that clusters 10 and 35 are enriched in CLL samples compared to HC. The three clusters express CD3, CD4, FOXP3, CD25, and CTLA4 which are characteristic of Treg populations. The proper identification of Treg cells require the co-expression of these markers, which can have variable expression depending on the subsets (37). The three clusters express high levels of PD-L1, LAG3. Clusters 10 and 35 express higher levels of PD1 compared to cluster 19, and cluster 10 shows the highest expression of KLRG1, representing fully activated and highly suppressive Tregs. In **Figure 10B**, the cluster 30, which is virtually not found in HC, is highly enriched in CLL samples. This cluster is composed of CD8^+^ T cells expressing high levels of inhibitory immune checkpoints KLRG1, LAG3, PD1 and CTLA4, characteristic for exhausted CD8^+^ T cells. By highlighting cluster 30 on the viSNE plot, we can clearly see that this cluster corresponds to the cells we identified as highly enriched in **Figure 10A** (red arrow). Concerning the monocytes, 3 clusters were enriched, with clusters 13 and 36 which represent patrolling monocytes expressing high levels of PD-L1 and LAG3 and being associated with CLL progression (**Figure 10C**). From these data, we concluded that the development of CLL is associated with the establishment of a very immunosuppressive microenvironment. In addition, the identified immunosuppressive cells in the CLL splenic microenvironment express high levels of immune checkpoints, particularly PD1 and LAG3. We next sought to validate if these immune checkpoints could represent potential therapeutic targets for an immunotherapeutic approach. To this end, we treated AT-TCL1 with blocking antibodies directed against PD1 and LAG3. This therapy led to a control of CLL development, with the restoration of a normal immuno-competent splenic microenvironment (2). Altogether, the study of the splenic CLL LME allowed us to better understand the immunosuppression found in CLL, to define clusters of cells highly enriched in CLL, and to identify potential targets for immunotherapy.

## Discussion

In addition to be costly, mass cytometry experiments tend to be time consuming (from panel design to sample staining, acquisition, and analysis). In order to make sure to fully benefit from this technology, all the steps must be carefully planned. Even though clearly limited compared to FC, contaminations between channels need to be scrupulously considered and eliminated as much as possible to avoid misinterpretation of the data. In addition to classical protein level quantification, mass cytometry offers a large range of detection of other biological parameters by the use of different probes. Post-translational modification, proteolysis, DNA synthesis, hypoxia, enzymatic activity, and chromatin modification can be assessed by this technique, opening up the possibilities for phenotypic analysis and discoveries of novel cell populations and unsuspected mechanism. However, the technology is currently limited to approximately the use of 50 channels, although the machines contains around 130 detectors. This is because the purity of some isolated metal isotopes is not high enough or it is not possible to couple those metals to antibodies/probes, although they could be detected by the mass cytometer. Effort is made to improve this pitfall, and the number of parameters analyzed in parallel will certainly increase in the coming years. The analysis of mass cytometry data is moving fast, with the development of new tools, not requiring bioinformatic skills. Mass cytometry is facing harsh competition with the development of fluorophore-based cytometers which can reach up to 30–50 parameters detected in parallel (such as the BDSymphony or bio-rad ZE5), and the emergence of spectral cytometers (Sony SP6800 and Cytek Aurora) positioning both technologies at the forefront of immunology research.

However, the advantages of mass cytometry still hold true, such as simpler panel design, multiplexing, minimal crosstalk among channel, no need of compensation. One main drawback of the technique, is the slower acquisition rate compared to classical FC, which can be overcome by performing a pre-enrichment of the cells of interest, in order to reduce the number of cells to acquire. However, it has to be noted that pre-enrichment can affect the final results.

In the recent years, a novel imaging technology has been developed (38), Imaging Mass Cytometry (IMC), which enables now the detection of up to 40 markers in a single imaging scan on tissue and tumor sections. This technology is provided by the same equipment as the mass cytometer, with adaptation for imaging. Complementary to mass cytometry, it allows the analysis of interaction between different cell types, but can also give insights on the activity of the cells, depending on their localization within the LME and interactions with other cells.

Mass cytometry represents a perfect asset for the study of LME in B cell malignancies. We applied this technology to the splenic microenvironment in the Eμ**-**TCL1 mouse model and identified a potential immunotherapeutic approach for the treatment of CLL. Other groups applied this technology to B malignancies, and these studies allowed to obtained new and innovative information concerning malignant B cells themselves or immune cells found in the LME. Maity et al., detected clusters of B cells specific to a subset of CLL patients bearing the R110 mutation in the particular light-chain allele IGLV3-21, and observed that these patients are phenotypically closer to unmutated (UM) patients, independently of their hypermutation status (6). In Follicular Lymphoma (FL), mass cytometry analysis allowed to understand that the malignant cells have a unique phenotype, not found in healthy donors. Contrary to what was shown based on gene expression profiling, the B malignant cells are not similar to germinal center (GC) B cells (3). By the study of immune cells in the context of Hodgkin Lymphoma, M. Shipp’s lab identified an enrichment of distinct regulatory T cells (Treg) populations with a T helper 1 (Th1) polarization phenotype (5). Finally, in FL, the presence of a specific T cell population expressing PD1 is associated with poor prognosis, whereas, the expression of PD1 on general T cells is not of prognostic value, highlighting the interest of having multiparametric analysis (4).

In conclusion, mass cytometry is an excellent tool to get phenotypical information at the single cell level, on protein expression, and the generalization of its use should lead to new discoveries, paving the road for the development of more specific and efficient therapies, targeting either directly the malignant cells or in the cells found in the LME.

## Material and Equipment

### Equipment

The Helios system (Cytometry by Time of Flight, CyTOF; Fluidigm) equipment was used for the data shown in this study. In addition, controls, for the expression of some markers, were acquired on conventional CytoFLEX (Beckam Coulter) flow cytometer.

### Cells and Reagents

All the reagents and cells used are described in *Stepwise Procedure for the Staining*.

## Data Availability Statement

The original contributions presented in this study are included in the article. Data presented in “Expected Results” were originally published in Blood2 [(2), Blood], and were reanalysed for the purpose of this article. Further inquiries can be directed to the corresponding authors.

## Author Contributions

SG, IF, MW, and AL wrote the manuscript. SG and MW performed the experiments, analyzed the data, and created the figures. GP, EG, and AC helped in writing, figures design, and for some experiments. EM, JP, and AL finalized the writing and supervised the team. All authors contributed to the article and approved the submitted version.

## Funding

This work was supported by grants from FNRS “Télévie” to SG, IF, MW, GP, and AL (7.4502.19, 7.4529.19, 7.6504.18, 7.4501.18, 7.4502.17, and 7.4503.19), from FNR Luxembourg to EG and JP (PRIDE15/10675146/CANBIO and INTER/DFG/16/11509946) and from Plooschter Projet.

## Conflict of Interest

The authors declare that the research was conducted in the absence of any commercial or financial relationships that could be construed as a potential conflict of interest.
